# Dipeptidyl peptidase-4 inhibitor cardiovascular safety in patients with type 2 diabetes, with cardiovascular and renal disease: a retrospective cohort study

**DOI:** 10.1038/s41598-021-95687-z

**Published:** 2021-08-17

**Authors:** Sheriza Baksh, Jiajun Wen, Omar Mansour, Hsien-Yen Chang, Mara McAdams-DeMarco, Jodi B. Segal, Stephan Ehrhardt, G. Caleb Alexander

**Affiliations:** 1 Department of Epidemiology, Johns Hopkins Bloomberg School of Public Health, Baltimore, MD 21205 USA; 2 Department of Health Policy and Management, Johns Hopkins Bloomberg School of Public Health, Baltimore, MD 21205 USA; 3 Center for Drug Safety and Effectiveness, Johns Hopkins University, Baltimore, MD 21205 USA; 4 Center for Health Services and Outcomes Research, Johns Hopkins University, Baltimore, MD 21205 USA; 5 Division of General Internal Medicine, Department of Medicine, Johns Hopkins Medicine, Baltimore, MD 21205 USA; 6 Johns Hopkins Bloomberg School of Public Health, 415 N. Washington Street, 2nd Floor, Baltimore, MD 21231 USA

**Keywords:** Epidemiology, Drug development, Endocrine system and metabolic diseases

## Abstract

Clinical trials investigating cardiovascular safety of dipeptidyl peptidase-IV inhibitors (DPP-4i) among patients with cardiovascular and renal disease rarely recruit patients with renal impairment, despite associations with increased risk for major adverse cardiovascular events (MACE). We investigated the risk of MACE associated with the use of DPP-4i among these high-risk patients. Using a new-user, retrospective, cohort design, we analyzed 2010–2015 IBM MarketScan Commercial Claims and Encounters for patients with diabetes, comorbid with cardiovascular disease and/or renal impairment. We compared time to first MACE for DPP-4i versus sulfonylurea and versus metformin. Of 113,296 individuals, 9146 (8.07%) were new DPP-4i users, 17,481 (15.43%) were new sulfonylurea users, and 88,596 (78.20%) were new metformin users. Exposure groups were not mutually exclusive. DPP-4i was associated with lower risk for MACE than sulfonylurea (aHR 0.84; 95% CI 0.74, 0.93) and similar risk for MACE to metformin (aHR 1.07; 95% CI [1.04, 1.16]). DPP-4i use was associated with lower risk for MACE compared to sulfonylureas and similar risk for MACE compared to metformin. This association was most evident in the first year of therapy, suggesting that DPP-4i is a safer choice than sulfonylurea for diabetes treatment initiation in high-risk patients.

## Introduction

Diabetes afflicts nearly 26 million people in the United States and is associated with considerable morbidity, mortality, and health care spending^1,2^. This statistic becomes especially concerning given that many patients with diabetes later develop diabetic complications such as diabetic retinopathy, diabetic ketoacidosis, and peripheral vascular disease. Additionally, patients with diabetes also often suffer from cardiovascular disease^3^. As such, patients and providers are left with a host of considerations for managing both the symptoms of diabetes and the associated comorbidities.

One important class of medicines to treat type 2 diabetes are the dipeptidyl peptidase-IV inhibitors (DPP-4i), which act by slowing the breakdown of glucagon-like peptide-1 (GLP-1), inhibiting glucagon release and increasing insulin release^4^. Since they were introduced in 2006, four DPP-4i have been approved by the FDA for stand-alone use or as part of fixed-dose combination products, and they rank third in utilization after metformin and sulfonylurea with 8% of antidiabetic drug prescriptions^5^.

DPP-4i were initially believed to be protective against major adverse cardiovascular events (MACE), evidenced through pre-market clinical trials^6^. Given high rates of cardiovascular disease among patients with diabetes^7,8^, as well as longstanding regulatory interest in the potential adverse cardiovascular events associated with diabetes treatments^9^, evidence of such a cardioprotective effect was of high regulatory, clinical and market importance. However, despite this early evidence from pre-market clinical pharmacology studies, postmarketing surveillance reports^10,11^ and data from Phase 4 clinical trials^12–14^ suggested a possible harmful association with MACE, specifically heart failure.

We examined the association between DPP-4i therapy and cardiovascular events using a large commercial claims database. We focused on individuals at elevated baseline risk, including those with history of cardiovascular disease as well as those with renal impairment, comparing the rates of adverse cardiovascular events among DPP-4i new users with that of metformin and sulfonylurea. The inclusion of individuals with renal impairment reflected increased regulatory interest in understanding cardiovascular risk in this patient population as evidenced in the two latest clinical trials studying the association of DPP-4i and cardiovascular outcomes^15,16^. Additionally, the results of the Saxagliptin Assessment of Vascular Outcomes Recorded in Patients with Diabetes Mellitus Thrombolysis in Myocardial Infarction 53 (SAVOR-TIMI 53) trial showed that an elevated urinary albumin to creatinine ratio was independently associated with increased risk for MACE^17^. We were interested in this elevated risk population, because if a discernable effect were present, it could mean additional cardiovascular risk from DPP-4i therapy for a population already at high susceptibility to cardiovascular events.

## Results

**Figure 1:**
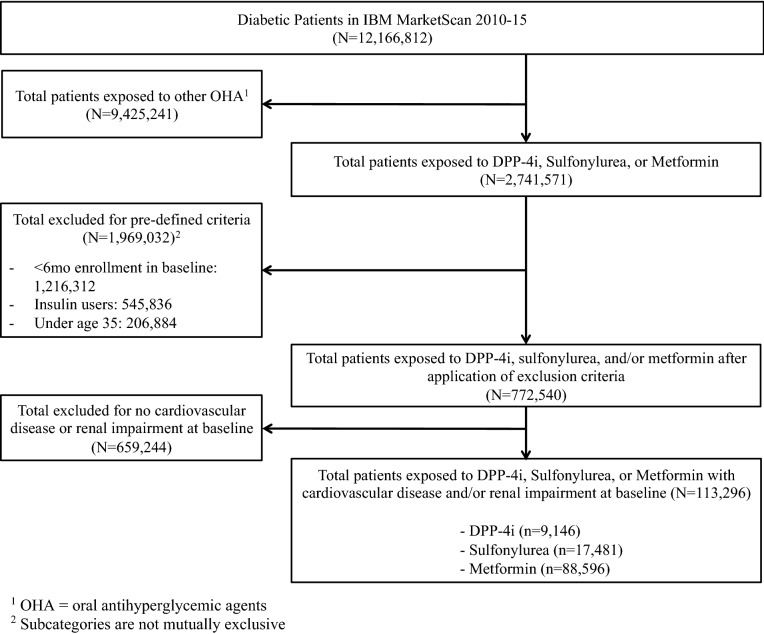
Cohort derivation and sample attrition after applying inclusion and exclusion criteria for retrospective cohort.

We identified 12,166,812 individuals with diabetes from 2010 to 2015. After applying our inclusion and exclusion criteria, there were a total of 113,296 individuals in our cohort (Fig. 1). Of these, 9146 (8.07%) were new users of DPP-4i, 17,481 (15.43%) initiated sulfonylureas, and 88,596 (78.20%) started metformin. Three percent of included individuals were new users of both DPP-4i and metformin, and 1524 (1.72%) were new users of both sulfonylureas and metformin.

Table 1 depict the demographic and clinical characteristics of DPP-4i users and their counterparts. For example, more sulfonylurea users (60.40%) were male compared to DPP-4i (58.51%) or metformin (52.17%) users. Approximately half of individuals in each exposure group had a cumulative exposure to the treatment of interest less than or equal to 6 months (DPP-4i: 54.05%; sulfonylureas: 56.28%; metformin: 53.65%). There were also several differences in baseline comorbidities, and corresponding medication use, between exposure groups. For example, rates of cardiovascular disease were higher among users of metformin than their counterparts, while kidney disease was more prevalent among users of sulfonylureas (17.22%) and DPP-4i (15.00%) than metformin (7.07%). Differences between users of DPP-4i, sulfonylurea, and metformin were not statistically significant after application of propensity score weighting.

**Table 1:** Baseline demographics and medical characteristics.

	DPP-4i (n = 9146)		Sulfonylureas (n = 17,481)		Metformin (n = 88,596)	
	N	%	N	%	N	%
Male sex	5351	58.51	10,559	60.40	46,224	52.17
**Age, years**						
35–44	877	9.59	2066	11.82	11,755	13.27
45–54	2815	30.78	5147	29.44	29,238	33.00
55–64	5454	59.63	10,268	58.74	47,603	53.73
**Cumulative exposure, months**						
< 6 months	4943	54.05	9838	56.28	47,534	53.65
6–12 months	2580	28.21	4326	24.75	23,869	26.94
12–18 months	848	9.27	1665	9.52	8713	9.83
> 18 months	775	8.47	1652	9.45	8480	9.57
**Comorbidities in baseline**						
Cardiovascular disease	8187	89.51	15,359	87.86	84,138	94.97
Kidney disease (chronic and acute)	1372	15.00	3011	17.22	6263	7.07
Cerebrovascular disease	1574	17.21	2931	16.77	14,391	16.24
Ischemic heart disease	3926	42.93	7374	42.18	35,242	39.78
Hypertension	7501	82.01	13,902	79.53	68,723	77.57
Eye disease	3149	34.43	5289	30.26	28,809	32.52
Renal disease^a^	4029	44.05	7243	41.43	34,718	39.19
Acute renal failure	465	5.08	1126	6.44	2118	2.39
Neuropathy	1320	14.43	2270	12.99	12,573	14.19
Nephropathy	559	6.11	1330	7.61	2455	2.77
**aDCSI score**						
0	5813	63.56	11,326	64.79	61,295	69.18
1	1203	13.15	2055	11.76	10,806	12.20
2	1358	14.85	2738	15.66	11,290	12.74
3 +	772	8.44	1362	7.79	5205	5.87
**Dual exposures**						
Sulfonylureas	0	0.00	0	0.00	1,524	1.72
Metformin	3451	37.73	1524	8.72	0	0.00
DPP-4 inhibitors	0	0.00	0	0.00	3451	3.90
**Concomitant baseline medications**						
ACE inhibitors	2022	22.11	3643	20.84	20,522	23.16
Angiotensin II receptor blockers	2380	26.02	3346	19.14	18,410	20.78
Antidepressants	2132	23.31	3378	19.32	24,832	28.03
Antiplatelets	2871	31.39	4729	27.05	28,182	31.81
Asthma medication	4599	50.28	7531	43.08	48,709	54.98
Benzodiazepines	1842	20.14	2905	16.62	20,485	23.12
Beta blockers	2832	30.96	5208	29.79	28,437	32.10
Blood thinners and anticoagulants	496	5.42	820	4.69	4924	5.56
Calcium channel blockers	1507	16.48	2762	15.80	13,754	15.52
Cardioselective beta blockers	940	10.28	1632	9.34	7767	8.77
Diuretics	1737	18.99	3016	17.25	19,258	21.74
Nitrates	807	8.82	1342	7.68	7924	8.94
Peripheral neuropathic treatments	688	7.52	944	5.40	6867	7.75
Statins	4699	51.38	7484	42.81	41,933	47.33
Thiazide diuretics	1875	20.50	3335	19.08	21,306	24.05

*aDCSI* adapted diabetes complications severity index.

^a^Includes diseases of kidneys, ureters, and bladders; inclusive of acute renal failure.

The median follow-up for the primary outcome was 160 days (interquartile range (IQR) 92–296 days) and there was no statistically significant difference in time-to-event distributions across the therapeutic classes examined. After propensity score weighting and adjusting for baseline demographics, clinical characteristics, and concomitant medications, the incidence rate for the primary outcome was statistically significantly less (30.20 per 1000 person-years) among new users of DPP-4i compared to sulfonylureas (Table 2). There was a non-statistically significant increase in the incidence rate for the primary outcome among new users of DPP-4i compared to sulfonylureas (91.25 per 1000 person-years vs. 79.46 per 1000 person-years). Among the secondary outcomes, the incidence rate for heart failure was statistically significantly less (4.07 per 1000 person-years vs. 7.56 per 1000 person-years) for new users of DPP-4i compared to sulfonylurea. The incidence rates for the secondary outcomes were not statistically significantly different between DPP-4i and metformin.

**Table 2:** Incidence rates of primary composite outcome, acute myocardial infraction, stroke, and heart failure among new users of DPP-4 inhibitors, sulfonylureas, and metformin.

Outcome, *n*	DPP-4 inhibitors	Sulfonylureas	Metformin
**Primary composite outcome**^1^	510	1301	4781
Total person years	5578	10,696	57,134
Rate per 1000 person years	91.25	121.63	79.46
Median [IQR] observation time, days	160 [92, 286]	144 [75, 288]	163 [93, 300]
Rate difference (95% CI)^2^	–	** − 30.20 [− 40.47, − 19.89]**	7.75 [− 0.45, 15.96]
**Acute myocardial infarction**	54	132	450
Total person years	5898	11,487	60,159
Rate per 1000 person years	9.16	11.49	7.06
Median [IQR] observation time, days	167 [98, 297]	161 [90, 308]	172 [100, 314]
Rate difference (95% CI)^2^	–	− 2.34 [− 5.45, 0.80]	1.68 [− 0.91, 4.22]
**Stroke**	141	268	1202
Total person years	5839	11,380	59,592
Rate per 1000 person years	24.15	23.55	20.17
Median [IQR] observation time, days	167 [97, 295]	159 [90, 305]	171 [99, 311]
Rate difference (95% CI)^2^	–	0.60 [− 4.31, 5.47]	3.98 [− 0.19, 8.14]
**Heart failure**	24	87	240
Total person years	5912	11,512	60,277
Rate per 1000 person years	4.06	7.56	3.98
Median [IQR] observation time, days	168 [98, 298]	161[90,308]	172 [100, 314]
Rate difference (95% CI)^2^	–	− **3.50 [**− **5.78,** − **1.21]**	0.08 [− 1.64, 1.82]

*IQR* interquartile range.

^1^Primary composite outcome includes myocardial infarction, cardiac arrest, coronary artery bypass, coronary angioplasty, heart failure, stroke, inpatient death.

^2^Incidence rate difference per 1000 person-years.

Bold values indicate statistically significant results.

After propensity score weighting and adjustment for baseline demographics, clinical characteristics, and concomitant medications, there was a statistically significant association between new use of DPP-4i and the primary outcome of MACE compared to sulfonylurea (adjusted hazard ratio (aHR) 0.84, 95% confidence interval (CI) 0.74–0.93). However, there was no statistically significant difference in risk of MACE among users of DPP-4i as compared with metformin (aHR 1.07, 95% CI 0.98–1.16) (Table 3). There were no statistically significant differences between sexes in the association between new use of DPP-4i and the primary outcome compared to sulfonylurea or metformin (Additional File 1, Supplementary Appendix 3).

**Table 3:** Hazard ratios for the association between DPP-4 inhibitor use and primary composite outcome, acute myocardial infraction, stroke, and heart failure compared to sulfonylureas and metformin.

Reference drug	Hazard ratios for DPP-4 inhibitors use			
	Primary composite outcome^c^	Acute myocardial infarction	Stroke	Heart failure
**Sulfonylureas**				
HR (95% CI)^a^	**0.77 [0.69, 0.93]**	0.81 [0.56, 1.07]	1.03 [0.84, 1.34]	**0.59 [0.35, 0.89]**
aHR (95% CI)^a^	**0.84 [0.74, 0.93]**	0.95 [0.72, 1.40]	1.08 [0.89, 1.35]	0.71 [0.35, 1.09]
**Metformin**				
HR (95% CI)^a^	0.98 [0.87, 1.08]	1.13 [0.92, 1.53]	1.12 [0.98, 1.34]	0.94 [0.62, 1.41]
aHR (95% CI)^b^	1.07 [0.98, 1.16]	1.32 [0.96, 1.81]	1.16 [0.98, 1.42]	1.19 [0.81, 1.81]

^a^Propensity score weighting only.

^b^Propensity score weighting and demographics, comorbidities, and concomitant medications as regressors and stratifiers.

^c^Primary composite outcome includes myocardial infarction, cardiac arrest, coronary artery bypass, coronary angioplasty, heart failure, stroke, inpatient death.

Bold values indicate statistically significant results.

There was a statistically significant association for heart failure in the propensity score weighted analysis comparing DPP-4i to sulfonylurea (HR 0.59, 95% CI 0.35–0.89); however, after adjusting for potential confounders, the association was attenuated and no longer statistically significant. DPP-4i was also not statistically significantly associated with acute myocardial infarction when compared to sulfonylureas (aHR 0.95, 95% CI 0.72–1.40) or metformin (aHR 1.32, 95% CI 0.96–1.81), nor was there a statistically significant association between DPP-4i and stroke when compared to sulfonylureas (aHR 1.08, 95% CI 0.89–1.35) or metformin (aHR 1.16, 95% CI 0.98–1.42).

When comparing the association between DPP-4i and the primary outcome by cumulative exposure strata, there were differences from the overall effect. In the comparison between new users of DPP-4i and sulfonylurea, the adjusted hazard ratio for the primary outcome was statistically significant for a cumulative exposure of < 6 months (aHR 0.85, 95% CI 0.71–0.93) and 6–12 months (aHR 0.76, 95% CI 0.70–0.93). However, statistical significance was not evident for cumulative exposure 12–18 months and > 18 months. This suggests that the overall aHR might be driven by differences in MACE risk seen in the first year after initial exposure. There were no differences between the overall and cumulative exposure stratum specific adjusted hazard ratios for MACE between new users of DPP-4i and metformin (Table 4).

**Table 4:** Hazard ratios for the association between DPP-4 inhibitor use and primary composite outcome, stratified by cumulative exposure.

Reference drug	Hazard ratios for DPP-4 inhibitors use		
	N_ref_	N_DPP-4i_	Hazard ratio [95% CI]
**Sulfonylureas**			
aHR^a^	17,481	9146	**0.84 [0.74, 0.93]**
Cumulative exposure < 6 months	9838	4943	**0.85 [0.71, 0.93]**
Cumulative exposure 6–12 months	4326	2580	**0.76 [0.70, 0.93]**
Cumulative exposure 12–18 months	1665	848	0.80 [0.90, 1.11]
Cumulative exposure > 18 months	1652	775	0.98 [0.92, 1.11]
**Metformin**			
aHR^a^	88,596	9146	1.07 [0.98, 1.16]
Cumulative exposure < 6 months	47,534	4943	1.10 [0.85, 1.14]
Cumulative exposure 6–12 months	23,869	2580	1.05 [0.92, 1.11]
Cumulative exposure 12–18 months	8713	848	0.97 [0.90, 1.12]
Cumulative exposure > 18 months	8480	775	1.16 [0.87, 1.13]

Primary composite outcome includes myocardial infarction, cardiac arrest, coronary artery bypass, coronary angioplasty, heart failure, stroke, inpatient death.

^a^Propensity score weighting and demographics, comorbidities, and concomitant medications as regressors and stratifiers.

Bold values indicate statistically significant results.

Results of analyses excluding individuals with acute renal failure (sulfonylureas: aHR 0.83, 95% CI 0.17–0.86; metformin: aHR 1.08, 95% CI 1.01–1.19) showed qualitatively similar results to those including these individuals (sulfonylureas: aHR 0.84, 95% CI 0.74–0.93; metformin: aHR 1.07, 95% CI 0.98–1.16). Similarly, results were not sensitive to changes in the latency period after the last dose of exposure for the primary analysis results, nor did they differ substantively when lagging the latency period by 7-days or 30-days (Additional File 1, Supplementary Appendix 4).

## Discussion

In this longitudinal study of commercial claims data for individuals with diabetes, having also cardiovascular disease and renal impairment, new use of DPP-4i was associated with a lower risk for MACE compared to sulfonylureas, and a comparable risk compared to metformin. This difference was most evident in the first year of use. New use of DPP-4i was not shown to be associated with the following individual components of MACE: heart failure, stroke, or acute myocardial infarction. These results contribute to the body of evidence examining the association of DPP-4i and MACE in individuals at higher risk for cardiovascular events.

Overall, our results corroborated those of previous clinical trials and observational studies of cardiovascular safety of DPP-4i. Our results showed no increased risk for MACE with the use of DPP-4i similar to the conclusions in the three completed clinical trials comparing DPP-4i and placebo^12,13,18^. However, unlike these trials our study population consisted of a high-risk group of individuals with established cardiovascular disease and renal impairment in a real-world setting with multiple comorbidities and concomitant medications. Additionally, our study allowed us to compare DPP-4i to other available therapies, namely sulfonylureas and metformin, showing a decreased risk for MACE when compared to sulfonylureas.

Three notable observational studies provide additional context to our results. First, a 2015 administrative claims study using the Taiwan National Health Insurance Research Database of individuals with diabetes and chronic kidney disease admitted for acute myocardial infarction also showed no increased risk for MACE when comparing individuals on sitagliptin to those not on sitagliptin^19^. Expanding on this approach, our study design included individuals with a multitude of cardiovascular conditions at baseline, making our results more generalizable to high-risk individuals with diabetes. The second study of 127,555 individuals which used the Italian Nationwide OsMed Health-DB database showed decreased risk in hospitalization for heart failure risk associated with DPP-4i compared to sulfonylurea (HR 0.78; 95% CI 0.62–0.97)^20^. This suggests that the increased risk for MACE in our comparison might be driven by differences in risk for heart failure. This is partially evident in the statistically significant unadjusted hazard ratio for heart failure as a secondary outcome (Table 3). Finally, a study of Medicare patients with diabetes compared cardiovascular risk of DPP-4i to sulfonylurea and thiazolidinediones and found no increased risk in individuals over age 65^21^. While our study was limited to a patient population under age 65, this study shows that our results are similar to those found in a study of older patients.

A major strength of our study was the identification of a high-risk cohort through the use of a large administrative database. The results of the SAVOR TIMI-53 trial showed that renal impairment was independently associated with adverse cardiovascular outcomes, even after adjusting for cardiovascular risk factors^17^. As such, our decision to restrict the study cohort to individuals with diabetes, comorbid with cardiovascular disease and renal impairment allowed us to hone in on a patient population identified by FDA as more appropriate for the study of this drug-event association. In a 2008 Guidance for Industry^9^, regulators noted that such patients are often excluded from pre-approval clinical trials, and recommended studies of the association between oral antihyperglycemic agents and MACE should include high-risk patients.

Our study also had several limitations. First, we included individuals using multiple therapies of interest (e.g., both DPP-4i and sulfonylureas) at baseline. While this may have led to potential misclassification, their exclusion would have resulted in a much smaller and less generalizable sample^22^. Second, our new-user design did not account for time-varying hazards for MACE, although the cumulative exposure stratified analysis suggested that the differences in risk of MACE between DPP-4i and sulfonylureas were most evident during the first year of exposure. Third, our analyses are subject to potential informative censoring due to a change in exposure status. However, our results were insensitive to the lagged latency period after the last day of exposure, and since most events occurred within the first year of exposure, extending the latency period past 30-days would have been unlikely to have changed our results and could have potentially led to misclassification. Finally, due to limitations in the administrative claims data, we were unable to assess mortality outside of the inpatient setting as a component of the primary composite outcome or as a competing risk. However, it is unlikely that death outside of the inpatient setting would occur more frequently in one exposure group than another in this cohort, potentially leading to biased results.

Our results provide evidence of decreased risk for MACE when comparing DPP-4i versus sulfonylurea in a commercially insured patient population with diabetes, comorbid with cardiovascular disease and renal impairment in the United States. Additionally, we found that there was no difference in risk of MACE for these individuals when comparing DPP-4i and metformin. Further studies are needed to determine differences in risk for individual components of the composite outcome, particularly heart failure. Finally, the decreased risk seen with DPP-4i use compared to sulfonylurea is more likely due to cardiovascular risk associated with sulfonylurea rather than protective effects of DPP-4i, as there was no difference in effect between DPP-4i and metformin, which carries little cardiovascular risk.

## Methods

### Study design and data source

We conducted a population-based, retrospective cohort study with a new-user design using data from IBM MarketScan Commercial Claims and Encounters from January 2010 through December 2015. MarketScan houses linked paid claims and encounter data from approximately 350 payers, covering more than 25 million individuals annually. The data consists of de-identified individual-level healthcare utilization data including demographic characteristics and information on inpatient and outpatient medical services and pharmacy claims issued.

### Cohort derivation

We identified individuals with type 2 diabetes as those with at least one prescription for an oral antihyperglycemic agent and either hemoglobin A1c greater than 6.5% twice, fasting glucose greater than 126 mg/dL twice on different days, random glucose > 200 mg/dL twice on different days, one inpatient diagnosis of diabetes (ICD-9(10) codes: 250x (E11.9), 357.2 (E11.42), 366.41 (E11.36), 362.01–362.07 (E11.3*)) or outpatient diagnosis for diabetes (ICD-9(10) codes: 250x (E11.9), 366.41 (E11.36), 362.01–362.07 (E11.3*)) twice on different days. We included individuals if they received at least one prescription for an FDA-approved DPP-4i, sulfonylurea, or metformin (Additional File 1, Supplementary Appendix 1). We assigned an index date as the date of first filled prescription for one of these products. The baseline period was defined as the six-month period preceding this. We used International Classification of Diseases, 9th and 10th Revisions (ICD-9/10) codes from inpatient and outpatient records to identify those with a history of cardiovascular disease and/or renal impairment (chronic kidney disease or acute renal failure) for inclusion into the study.

We excluded individuals based on: (1) no continuous medical or pharmacy enrollment in the six-month baseline period; (2) below the age of 35; (3) insulin use at baseline; (4) end-stage renal disease in the baseline period; (5) less than 12-weeks of exposure to index treatment; or (6) treatment with oral antihyperglycemic agents in the 6-month baseline period. We followed individuals until the first of the following occurrences: (1) 14-days after the last date of exposure; (2) switch to or addition of anti-hyperglycemic treatment that was not DPP-4i, sulfonylurea, or metformin; (3) end in medical or pharmacy enrollment; (4) first date of either a major adverse cardiovascular event; or (5) study end date of 31 December 2015.

### Definition of exposure

We assigned individuals to an exposure group based on their first prescription of DPP-4i, sulfonylurea, or metformin. In the event of multiple drug class prescriptions on the index date, individuals were counted in all relevant exposure groups. Due to the large number of individuals on multiple antihyperglycemic agents, particularly metformin in combination with other drug classes, dropping these individuals would have resulted in a considerably smaller sample size, reducing statistical power as well as the generalizability of our results.

### Definition of outcome

We defined our primary composite outcome as the first of any of the following events: myocardial infarction, cardiac arrest, coronary artery bypass graft, coronary angioplasty, heart failure, and stroke. We did not examine all-cause mortality outside of the inpatient setting as an outcome, because information on these deaths were not available for our dataset. Our secondary outcomes were acute myocardial infarction, stroke, and heart failure. We used validated algorithms to identify myocardial infarction^23^, cardiac arrest^24^, coronary artery bypass graft^23^, coronary angioplasty^23^, heart failure^25^, and stroke^26^.

### Definition of covariates

We used the peer-reviewed literature^19,27–29^, clinical guidelines^30–34^ and expert opinion^9,35^ in order to identify key covariates of interest. We assessed possible confounding due to patient age and sex; cardiovascular risk factors including hypertension and hypercholesterolemia, diabetic complications, diabetic severity at baseline as measured by the Adjusted Diabetes Comorbidities Severity Index (aDCSI)^36–38^, and other common comorbidities such as cerebrovascular disease, ischemic heart disease, asthma, and rheumatoid arthritis. Comorbidities coded under ICD-9 were identified using the Clinical Classification Software (CCS) developed by the Agency for Healthcare Research and Quality^39^. Additionally, we used National Drug Codes to assess for possible confounding due to concomitant medications including statins, angiotensin converting enzymes (ACE) inhibitors, platelet aggregation inhibitor, calcium channel blockers, angiotensin II receptor blockers, beta-blockers, diuretics, nitrates, and inhaled corticosteroids.

### Propensity score

We used the Toolkit for Weighting and Analysis of Nonequivalent Groups (*twang)* package developed by the RAND Corporation^40^ to compute the propensity scores and associated weights used in the analysis to balance the covariates between exposure groups. To do so, we identified all available covariates without an association to the exposure that were associated to the primary outcome in order to increase precision^41^ (Additional File 1, Supplementary Appendix 2). Next, we used generalized boosted regression models to optimize the selection of covariates for the propensity score calculation, allowing for propensity scores estimation in the presence of multiple exposure groups. We used the standardized mean difference (SMD) to measure balance of covariates before and after weighting. Propensity score weighting reduced the SMD from a maximum of 0.23 to less than 0.01. We used the average treatment effect on the treated (ATT) propensity score weights to estimate the treatment effect of DPP-4i.

### Statistical analysis

We identified a baseline period of 6 months prior to the first filled prescription of the exposure group. Using chi-square statistics for categorical covariates and the Kruskal–Wallis test for continuous covariates, we compared differences at baseline between new users of DPP-4i, sulfonylurea, and metformin. We used exact Poisson tests to compare absolute differences in incidence rates for the primary and secondary outcomes for new users of DPP-4i compared to those of sulfonylurea and metformin. Additionally, we checked for differences in duration of exposure to treatment distributions using a Kolmogorov–Smirnov test. Using the propensity score weights described above, we calculated weighted crude and adjusted Cox proportional hazards for the association between new use of DPP-4i and the primary and secondary outcomes compared to new use of sulfonylureas and metformin. For the adjusted hazard ratios, we included indicators for age, sex, baseline comorbidities, and concomitant medications. Additionally, we checked for covariates that violated the proportional hazards assumption by plotting the scaled Schoenfeld residuals. These covariates were added to the Cox proportional hazards model as stratifying variables. We also used spline intervals every 6 months to address potential violations of the proportional hazard assumption. Finally, we included an indicator variable for individuals included in multiple exposure groups and an indicator variable for cumulative exposure, which was defined as the number of days exposed to the exposure group drug. We stratified the analysis by the cumulative exposure variable. All analyses were conducted in R, version 3.3.3.

### Sensitivity analyses

To check the robustness of our results, we first assessed possible sensitivity of our results to the latency of the period after drug discontinuation. We followed individuals for 14-, 7-, and 30-days after the last day of exposure to drug. Next, we recalculated the primary analysis without individuals with acute renal failure to determine whether our results were sensitive to the inclusion of those individuals. Finally, we compared the association of DPP-4i and the primary outcome between cumulative exposure strata and sex strata to assess possible effect modification.

The study was exempted from review by a Johns Hopkins Institutional Review Board, and all research was performed in accordance with relevant guidelines/regulations. Our study utilized commercial claims data through IBM Marketscan Research Databases. As such, we did not obtain informed consent. This was in accordance with local laws and regulations, and aligned with the terms of use from IBM Marketscan Research Databases.

### Ethics approval

The study was exempted from review by a Johns Hopkins Institutional Review Board.

## Supplementary Information

Supplementary Information.

## Data Availability

The data that support the findings of this study are available from IBM MarketScan Research Databases but restrictions apply to the availability of these data, which were used under license for the current study, and so are not publicly available. Data are however available from the authors upon reasonable request and with permission of IBM MarketScan Research Databases.
