# Supplementary material for: Dipeptidyl peptidase-4 inhibitor cardiovascular safety in patients with type 2 diabetes, with cardiovascular and renal disease: a retrospective cohort study
Source: Sci Rep. 2021 Aug 17;11:16637. doi: 10.1038/s41598-021-95687-z (PMC8371013; doi:10.1038/s41598-021-95687-z)
Supplement: Supplementary file 1 — Supplementary Information. [file 41598_2021_95687_MOESM1_ESM.docx]

**SUPPLEMENTARY MATERIAL**

**Appendix 1.** Categorization of drugs into exposure groups

*FDA Approved DPP-4 Inhibitors*

alogliptin

alogliptin and metformin

alogliptin and pioglitazone

linagliptin

linagliptin and empagliflozin

linagliptin and metformin

linagliptin and metformin extended release

sitagliptin

sitagliptin and metformin

sitagliptin and metformin extended release

saxagliptin

saxagliptin and metformin extended release

*FDA Approved Sulfonylureas*

chlorpropamide

glimepiride

glimepiride and pioglitazone hydrochloride

glimepiride and rosiglitazone maleate

glipizide

glipizide extended release

glyburide

glyburide and metformin hydrochloride

tolazamide

tolbutamide

*FDA Approved Biguanides**

metformin hydrochloride

metformin hydrochloride extended release

| **Appendix 2.** Covariates used in propensity score model and adjusted Cox proportional hazards model | | | |  |
| --- | --- | --- | --- | --- |
| **Covariate** | **Included in Propensity Score Model** | **Included in adjusted Cox PH Model** | | |
|  |  | **Covariate** | **Stratifier** | |
| *Demographics* |  |  |  | |
| Male | **X** |  | **X** | |
| Age | **X** |  | **X** | |
| Location (Region) |  |  |  | |
|  |  |  |  | |
| *Cumulative Exposure* |  |  | **X** | |
|  |  |  |  | |
| *Comorbidities in Baseline* |  |  |  | |
| MACE | **X** | **X** |  | |
| Kidney disease | **X** | **X** |  | |
| Cerebrovascular disease | **X** |  | **X** | |
| Congestive heart failure |  |  |  | |
| Ischemic heart disease | **X** | **X** |  | |
| Hypertension | **X** | **X** |  | |
| Retinopathy |  |  |  | |
| Eye disease | **X** | **X** |  | |
| Renal disease | **X** |  | **X** | |
| Acute renal failure |  |  |  | |
| Atrial fibrilation |  |  |  | |
| Neuropathy | **X** | **X** |  | |
| Nephropathy | **X** | **X** |  | |
| aDCSI Score | **X** |  | **X** | |
|  |  |  |  | |
| *Dual Exposures* |  |  |  | |
| Sulfonylureas |  |  |  | |
| Metformin |  | **X** |  | |
| DPP-4 inhibitors |  | **X** |  | |

(Continued)

| **Appendix 2 (cont'd). Covariates used in propensity score model and adjusted Cox proportional hazards model** | | | | |
| --- | --- | --- | --- | --- |
| **Covariate** | **Included in Propensity Score Model** | **Included in adjusted Cox PH Model** | |  |
|  |  | **Covariate** | **Stratifier** |  |
| *Concomitant Medications at Baseline* |  |  |  |  |
| α-Glucosidase inhibitors |  |  |  |  |
| ACE inhibitors | **X** | **X** |  |  |
| alpha agonists | **X** |  |  |  |
| analgesics |  |  |  |  |
| angiotensin II receptor  blockers | **X** | **X** |  |  |
| anti Veg-F |  |  |  |  |
| anticoagulants | **X** |  |  |  |
| antidepressants | **X** | **X** |  |  |
| antiplatelets | **X** |  | **X** |  |
| aspirin | **X** |  |  |  |
| asthma medication | **X** | **X** |  |  |
| benzodiazepines | **X** | **X** |  |  |
| beta blockers | **X** |  | **X** |  |
| beta blockers (ophthalmic) |  |  |  |  |
| bile acid sequestrants |  |  |  |  |
| biologic response modifiers |  |  |  |  |
| blood thinners and anticoagulants | **X** | **X** |  |  |
| Bronchodilators | **X** |  |  |  |
| calcium channel blockers | **X** | **X** |  |  |
| carbonic anhydrase inhibitors |  |  |  |  |
| cardioselective beta blockers | **X** | **X** |  |  |
| cholinergics |  |  |  |  |
| disease-modifying antirheumatic  drugs |  |  |  |  |
| diuretics | **X** | **X** |  |  |
| erythropoietan | **X** |  |  |  |
| fibrates |  |  |  |  |
| GLP-1 agonists |  |  |  |  |
| hormone replacement therapy |  |  |  |  |
| inhaled steroids | **X** |  |  |  |
| leukotrine modifiers | **X** |  |  |  |

(Continued)

| **Appendix 2 (cont'd).** Covariates used in propensity score model and adjusted Cox proportional hazards model | | | |  |
| --- | --- | --- | --- | --- |
| **Covariate** | **Included in Propensity Score Model** | **Included in adjusted Cox PH Model** | | |
|  |  | **Covariate** | **Stratifier** | |
| *Concomitant Medications at Baseline* |  |  |  | |
| loop diuretics | **X** |  |  | |
| MAOI | **X** |  |  | |
| meglitinides |  |  |  | |
| niacin | **X** |  |  | |
| nitrates | **X** | **X** |  | |
| NSAIDs |  |  |  | |
| ophthalmic drugs |  |  |  | |
| oral corticosteroids | **X** |  |  | |
| peripheral neuropathic  treatments | **X** | **X** |  | |
| phosphodiesterase-4  inhibitors |  |  |  | |
| potassium sparing  diuretics | **X** |  |  | |
| prostaglandins | **X** |  |  | |
| SGLT-2 inhibitors |  |  |  | |
| SNRI | **X** |  |  | |
| SSRI | **X** |  |  | |
| statins | **X** |  | **X** | |
| theophyllines |  |  |  | |
| thiazide diuretics | **X** | **X** |  | |
| thiazolidinediones |  |  |  | |
| tricyclic antidepressants | **X** |  |  | |
| vasodilators | **X** |  |  | |

| **Appendix 3.** Hazard ratios for the association between DPP-4 inhibitor use and primary composite outcome^1^, stratified by sex | | | |
| --- | --- | --- | --- |
|  | **Hazard Ratios for DPP-4 Inhibitors Use** | | |
| **Reference Drug** | **N_ref_** | **N_DPP-4i_** | **Hazard Ratio [95% CI]** |
| Sulfonylureas |  |  |  |
| aHR^2^ | 17,481 | 9,146 | **0.84 [0.74, 0.93]** |
| Male | 10,559 | 5,351 | **0.86 [0.74, 0.99]** |
| Female | 6,922 | 3,795 | **0.79 [0.65, 0.98]** |
| Metformin |  |  |  |
| aHR^2^ | 88,596 | 9,146 | 1.07 [0.98, 1.16] |
| Male | 46,224 | 5,351 | 1.05 [0.93, 1.19] |
| Female | 42,372 | 3,795 | 1.10 [0.92, 1.32] |
| ^1^ Primary composite outcome includes myocardial infarction, cardiac arrest, coronary artery bypass, coronary angioplasty, heart failure, stroke, death | | | |
| ^2^ Propensity score weighting and demographics, comorbidities, and concomitant medications as regressors and stratifiers | | | |

| **Appendix 4.** Hazard ratios for the association between DPP-4 inhibitor use and primary composite outcome, showing sensitivity to latency after drug discontinuation | | | |
| --- | --- | --- | --- |
|  | **Hazard Ratios for DPP-4 Inhibitors Use** | | |
| **Reference Drug** | **14-day lag censor** | **7-day lag censor** | **30-day lag censor** |
| Sulfonylureas |  |  |  |
| aHR (95% CI)^1^ | **0.84 [0.74, 0.93]** | **0.85 [0.69, 0.91]** | **0.81 [0.73, 0.90]** |
| Metformin |  |  |  |
| aHR (95% CI)^1^ | 1.07 [0.98, 1.16] | 1.09 [1.00, 1.23] | 1.05 [0.90, 1.15] |
| ^1^ Propensity score weighting and demographics, comorbidities, and concomitant medications as regressors and stratifiers | | | |

| **Appendix 5. Hazard ratios for the association between DPP-4 inhibitor use and primary composite outcome, showing sensitivity to individuals with more than one exposure group** | | |
| --- | --- | --- |
|  | **Hazard Ratios for DPP-4 Inhibitors Use** | |
| **Reference Drug** | **Allowing for dual exposure** | **Disallowing dual exposure** |
| Sulfonylureas |  |  |
| HR (95% CI)^1^ | **0.77 [0.69, 0.93]** | **0.76 [0.69, 0.92]** |
| aHR (95% CI)^2^ | **0.84 [0.74, 0.93]** | **0.84 [0.73, 0.93]** |
| Metformin |  |  |
| HR (95% CI)^1^ | 0.98 [0.87, 1.08] | 1.00 [0.89, 1.11] |
| aHR (95% CI)^2^ | 1.07 [0.98, 1.16] | 1.10 [1.00, 1.20] |
| ^1^ Propensity score weighting only | | |
| ^2^ Propensity score weighting, spline terms for cumulative exposure, and demographics, comorbidities, and concomitant medications as regressors and stratifiers | | |

| **Appendix 6.** Pre- and Post-weighting distribution of baseline covariates across exposure groups | | | | | | | | | |
| --- | --- | --- | --- | --- | --- | --- | --- | --- | --- |
|  | DPP4i | Sulfonylurea | | | | Metformin | | | |
|  |  | Pre-weighting | | Post-weighting | | Pre-weighting | | Post-weighting | |
|  | Mean | Mean | p-value | Mean | p-value | Mean | p-value | Mean | p-value |
| Male | 0.59 | 0.60 | **<.001** | 0.59 | 0.93 | 0.52 | **<.001** | 0.58 | 0.91 |
| Age Group | 0.10 | 0.12 | **<.001** | 0.10 | 0.98 | 0.13 | **<.001** | 0.10 | 0.99 |
| Cardiovascular disease | 0.10 | 0.12 | **<.001** | 0.11 | 0.96 | 0.05 | **<.001** | 0.10 | 0.85 |
| Kidney Disease | 0.85 | 0.83 | **<.001** | 0.85 | 0.94 | 0.93 | **<.001** | 0.85 | 0.92 |
| Cerebrovascular disease | 0.83 | 0.83 | 0.36 | 0.83 | 0.87 | 0.84 | **0.02** | 0.83 | 0.95 |
| Ischemic heart disease | 0.57 | 0.58 | 0.24 | 0.57 | 0.84 | 0.60 | **<.001** | 0.57 | 0.90 |
| Hypertension | 0.18 | 0.20 | **<.001** | 0.18 | 0.97 | 0.22 | **<.001** | 0.18 | 0.94 |
| Eye disease | 0.66 | 0.70 | **<.001** | 0.66 | 0.87 | 0.67 | **<.001** | 0.66 | 0.95 |
| Renal disease | 0.56 | 0.59 | **<.001** | 0.56 | 0.77 | 0.61 | **<.001** | 0.56 | 0.88 |
| Neuropathy | 0.86 | 0.87 | **<.001** | 0.86 | 0.93 | 0.86 | 0.53 | 0.86 | 0.92 |
| Nephropathy | 0.94 | 0.92 | **<.001** | 0.94 | 0.98 | 0.97 | **<.001** | 0.94 | 0.87 |
| aDCSI score | 0.64 | 0.65 | **<.001** | 0.64 | 0.99 | 0.69 | **<.001** | 0.64 | 1.00 |
| Benzodiazepines | 0.80 | 0.83 | **<.001** | 0.80 | 0.95 | 0.77 | **<.001** | 0.80 | 0.93 |
| Antidepressant | 0.77 | 0.81 | **<.001** | 0.77 | 0.92 | 0.72 | **<.001** | 0.77 | 0.91 |
| ACE inhibitors | 0.78 | 0.79 | **0.02** | 0.78 | 0.92 | 0.77 | **0.02** | 0.78 | 0.95 |
| Antiplatelets | 0.69 | 0.73 | **<.001** | 0.69 | 0.91 | 0.68 | 0.41 | 0.69 | 0.94 |
| Angiotensin II receptor blockers | 0.74 | 0.81 | **<.001** | 0.74 | 0.78 | 0.79 | **<.001** | 0.74 | 0.97 |
| Beta blockers | 0.69 | 0.70 | 0.05 | 0.69 | 0.98 | 0.68 | **0.03** | 0.69 | 0.97 |
| Cardioprotective beta blockers | 0.90 | 0.91 | **0.01** | 0.90 | 0.86 | 0.91 | **<.001** | 0.90 | 0.98 |
| Diuretics | 0.81 | 0.83 | **<.001** | 0.81 | 0.94 | 0.78 | **<.001** | 0.81 | 0.92 |
| Nitrates | 0.91 | 0.92 | **<.001** | 0.91 | 0.87 | 0.91 | 0.70 | 0.91 | 0.87 |
| Statins | 0.49 | 0.57 | **<.001** | 0.49 | 0.87 | 0.53 | **<.001** | 0.49 | 0.88 |
| Peripheral neuropathic agents | 0.92 | 0.95 | **<.001** | 0.93 | 0.87 | 0.92 | 0.44 | 0.93 | 0.89 |
| Calcium channel blockers | 0.84 | 0.84 | 0.15 | 0.83 | 0.96 | 0.84 | **0.02** | 0.84 | 0.92 |
| Thiazide diuretics | 0.79 | 0.81 | **0.01** | 0.79 | 0.95 | 0.76 | **<.001** | 0.79 | 0.86 |
| Asthma medication | 0.50 | 0.57 | **<.001** | 0.50 | 0.79 | 0.45 | **<.001** | 0.50 | 0.98 |
| Blood thinners and anticoagulants | 0.95 | 0.95 | **0.01** | 0.95 | 0.94 | 0.94 | 0.59 | 0.95 | 0.95 |
